# Comparison of Losartan and Furosemide Interaction with HSA and Their Influence on HSA Antioxidant Potential

**DOI:** 10.3390/ph15050499

**Published:** 2022-04-19

**Authors:** Wojciech Rogóż, Jadwiga Pożycka, Aleksandra Owczarzy, Karolina Kulig, Małgorzata Maciążek-Jurczyk

**Affiliations:** Department of Physical Pharmacy, Faculty of Pharmaceutical Sciences in Sosnowiec, Medical University of Silesia in Katowice, 40-055 Katowice, Poland; wrogoz@sum.edu.pl (W.R.); jpozycka@sum.edu.pl (J.P.); aowczarzy@sum.edu.pl (A.O.); kkulig@sum.edu.pl (K.K.)

**Keywords:** losartan, furosemide, HSA, antioxidant, nanoITC, spectroscopy

## Abstract

Serum albumin (HSA) is the most important protein in human body. Due to the antioxidant activity, HSA influences homeostasis maintenance and transport of drugs as well as other substances. It is noteworthy that ligands, such as popular drugs, modulate the antioxidant activity of HSA. The aim of this study was to analyze the influence of losartan (LOS) and furosemide (FUR) on HSA antioxidant properties as well as the interaction between these drugs and protein using calorimetric and spectroscopic methods. LOS and FUR showed the high affinity for human serum albumin, and the binding reactions between them were spontaneous and exothermic. LOS and FUR, separately and together in the system, have no significant impact on the secondary HSA structure; however they have significant impact on the tertiary HSA structure. LOS and FUR mixed with HSA have the ability to scavenge free radicals, and the ligand(s)–HSA interactions were synergistic.

## 1. Introduction

Human serum albumin (HSA) is a very important protein in human blood plasma. Due to its properties, HSA influences the maintenance of homeostasis, the transport of drugs as well as other substances. An important function of HSA is also participation in adjusting the level of free radicals (especially in the blood) [1]. Both HSA and also glutathione, uric acid or coenzyme Q10 are responsible for endogenous antioxidant mechanisms that maintain the level of free radicals [2]. Free radicals are specific molecules with an unpaired electron on the valence shell. Due to chemical reactivity, they react with proteins, as well as lipids, sugars and nucleic acids. Free radicals take part in immune reactions and pathogen damage, and they are also an important element of signaling pathways in the human body. Unfortunately, a high level of free radicals is a common feature of many diseases, including civilization diseases, as well as the aging processes [3,4,5].

Mutual interactions between various substances such as drugs can have a significant impact on their biological activity [6]. Chemicals bound with HSA modulate its antioxidant activity as well as modify HSA properties and structure [7,8]. The interaction between biologically active substances are antagonistic, synergistic and additive. In the first case the effect of the simultaneous action of the analyzed components of the mixture is smaller than when they are separate. In the case of synergistic interaction, the effect is bigger while the additive effect of the interaction between substances is observed when the components of the mixture do not affect each other [6,9].

**Figure 1:**
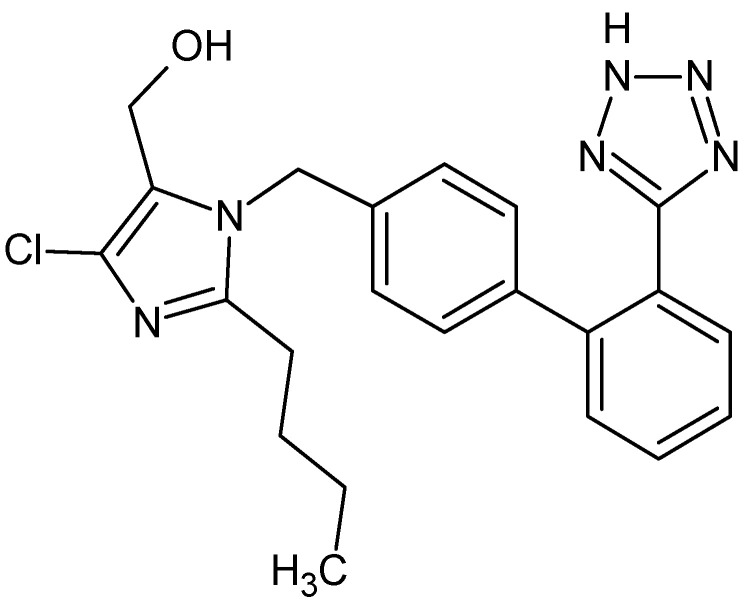
Structural formula of losartan (LOS) (ChemSketch 12.1.0.31258).

**Figure 2:**
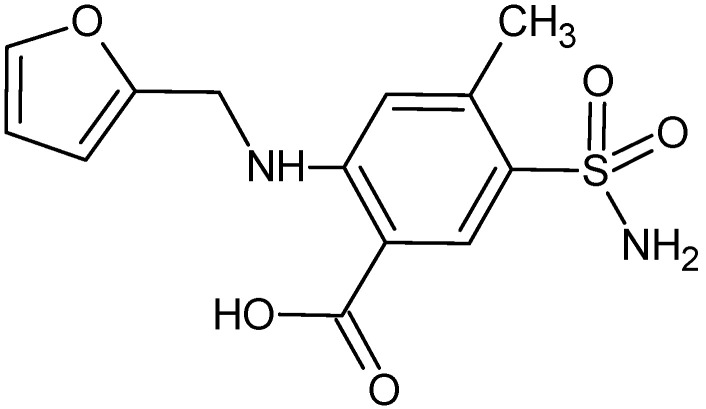
Structural formula of furosemide (FUR) (ChemSketch 12.1.0.31258).

Blood pressure medications are the most common used drugs, and they include among others angiotensin II receptor blockers (i.e., losartan) and diuretics (i.e., furosemide). Losartan (LOS, Figure 1) is used to treat many diseases such as hypertension, diabetic nephropathy, heart failure and isolated systolic hypertension (ISH) [10,11,12]. Puskarich et al. studied that LOS can also has an influence on hospitalized patients with COVID-19 [13]. Furosemide (FUR, Figure 2) is used to treat edematous states as well as also hypertension [14,15] and simultaneous application with LOS may have clinical consequences [16,17,18]. Based on the literature it is known that the main binding site for LOS as well as LOS metabolite (E 3174) is the HSA surface and Sudlow’s site II, respectively [11], while for FUR on the HSA surface, there are high and low affinity binding sites [19,20].

The main aim of this study was to compare losartan and furosemide interaction with human serum albumin as well as their influence on HSA structure and antioxidant activity. This work presents an innovative practical approach for the application of microcalorimetry in studying drugs—albumin interaction in combination with the spectroscopic methods for the analysis of antioxidant albumin properties. This novelty aspect of the research helps to optimize several drug selections in multidrug therapy not only in terms of their simultaneous use but also in terms of regulating the level of free radicals in the human body. 

## 2. Results and Discussion

### 2.1. Calorimetric and Spectroscopic Analysis of LOS and FUR Interaction with Human Serum Albumin

**Figure 3:**
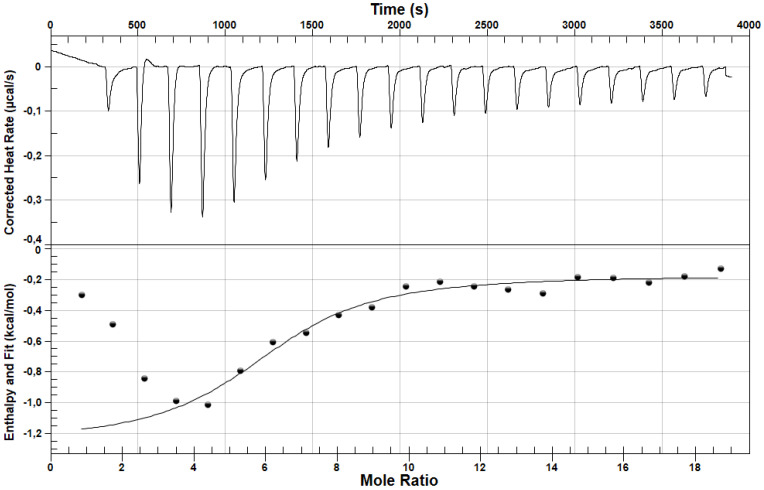
A nanoITC thermogram of albumin in the presence of LOS (LOS:HSA ~1:1 ÷ ~19:1 molar ratio); the upper figure shows the raw heat data obtained from the consecutive injections while the lower figure presents binding isotherm created by plotting areas of the heat peak in relation to the molar ratio of losartan to albumin. The lines present the best fit of the models used. T = 298 [K]. NanoAnalyze Data Analysis Version 3.10.0 (TA Instruments, New Castle, DE, USA).

**Figure 4:**
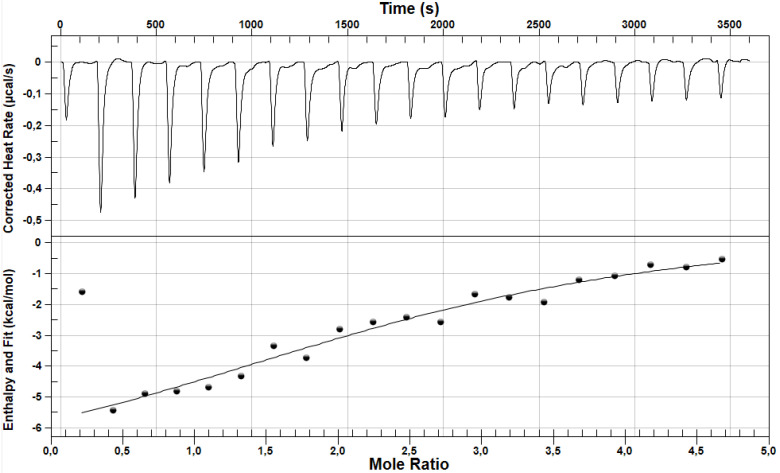
A nanoITC thermogram of albumin in the presence of FUR (FUR:HSA ~0.2:1 ÷ ~4.7:1 molar ratio); the upper figure shows the raw heat data obtained from the consecutive injections while the lower figure presents binding isotherm created by plotting areas of the heat peak in relation to the molar ratio of furosemide to albumin. The lines present the best fit of the models used. T = 298 [K]. NanoAnalyze Data Analysis Version 3.10.0 (TA Instruments, New Castle, DE, USA).

With the use of nanoITC, based on the analysis of thermograms and thermodynamic parameters, the interactions between losartan (LOS) and furosemide (FUR) were studied. Figure 3 presents a nanoITC thermogram of LOS in the complex with albumin while Figure 4 presents a nanoITC thermogram of FUR in the complex with albumin at molar ratios ranges LOS:HSA ~1:1 ÷ ~19:1 and FUR:HSA ~0.2:1 ÷ ~4.7:1, respectively. The obtained thermodynamic parameters characterizing the interaction between the analyzed drugs and HSA are presented in Table 1. 

NanoITC data help to analyze drugs interaction with HSA and to determine in one step the thermodynamic parameters accompanying the bonds formation as well as their nature. On the basis of data presented in Table 1, it can be concluded that both LOS and FUR can bind to HSA, as well as the affinity of both drugs (LOS and FUR) for HSA is relatively high. Interactions between LOS/FUR and HSA were previously analyzed based on spectroscopic methods, especially the spectrofluorometric, by Bojko [19], Moeinpour [20], Yasseen [21], Szkudlarek [22] and others, and there are not a lot of papers concerning the nanoITC analysis of LOS and FUR binding with protein [23]. Analyzing the LOS-HSA complex, the researchers obtained association constants values of the same order. Moeinpour et al. calculated association constant K_a_ = 8.9 × 10^4^ M^−1^, while Szkudlarek et al. K_a_ = (8.1 ± 0.41) × 10^4^ M^−1^ and K_a_ = (8.6 ± 0.11) × 10^4^ M^−1^ (λ_ex_ 275 nm; T = 310 K, Scatchard and Klotz method, respectively) [20,22]. In the case of the association constant for the FUR-HSA complex, the value calculated by Yasseen was K_a_ = 1.4 × 10^4^ M^−1^ [21]. Using ITC microcalorimeter and fluorescence spectrophotometer, the association constants by Zaidi were K_a_ = (8.2 ± 0.41) × 10^4^ M^−1^ and K_a_ = 4.09 × 10^5^ M^−1^, respectively [23]. As it can be concluded, the results obtained by different methods, sometimes under different environmental conditions, are not always fully compatible with each other. For this reason, the analyses of drugs affinity with proteins using precise techniques are of great importance. NanoITC is a very modern and extremely precise technique, and it provides the highest reliability and repeatability of results. The value of the stoichiometric binding sites number (n) approximately equals to six for LOS and between one and two for FUR, suggesting that more than one molecule of drugs (LOS and FUR) bind to one molecule of HSA. Although the spectroscopic methods allowed to obtain only one LOS molecule bound with HSA molecules [20,22], nanoITC measurements due to the high sensitivity provide data concerning not only the highest affinity but also the lowest where the interactions drug–albumin are weak. In a similar way to the present study, Zaidi et al. using the ITC calorimetric technique identified two FUR high and low affinity binding sites on HSA molecule, while with the use of the spectroscopic method, the value of n parameter was one, suggesting one class of binding sites [23]. 

Due to the negative value of Gibbs free energy change (ΔG < 0), the binding reaction of HSA with LOS as well as FUR was spontaneous. The enthalpy change for both analyzed reactions (ligand–protein) was negative (ΔH < 0), and this proves the exoenergetic nature of reaction accompanied by the release of energy into the environment. The mutual relation between ΔH and ΔS values is very important, and it allows to determine the type of non-covalent bond between the ligand and the protein. Based on the data collected in Table 1, negative or close to zero ΔH and positive ΔS in case of LOS was obtained. According to the data presented by Ross et al., this phenomenon means the presence of ionic bonds (electrostatic forces) [24,25,26]. In case of interaction between FUR and HSA the value of ΔH is smaller, and ΔS is less than zero. It suggests the dominance of van der Waals forces and hydrophobic interaction. At same time, there is the possibility of occurrence hydrogen bonds [23,25,27,28,29].

**Figure 5:**
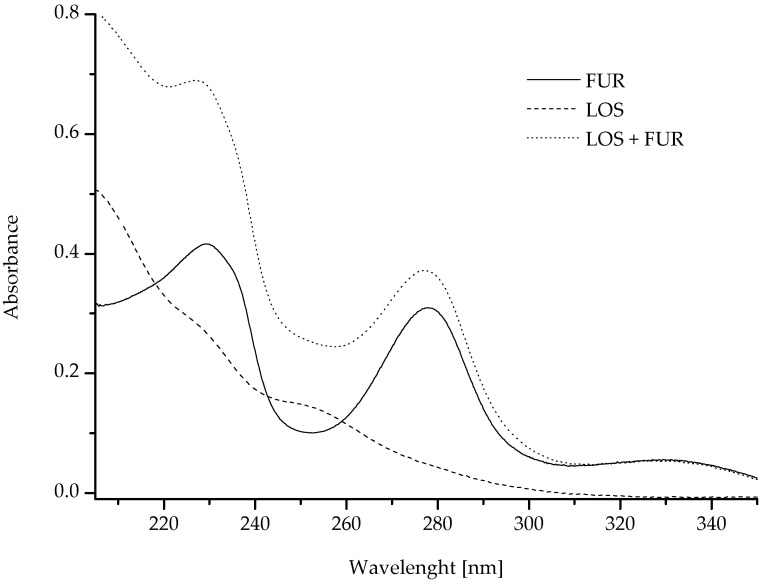
The absorption spectra of LOS, FUR and drugs mixture ([LOS] = [FUR] = 1 × 10^−5^ M; molar ratio LOS:FUR 1:1).

There are countless opportunities for the mutual interaction between various drugs. Some are commonly known and therefore easy to avoid in therapy. Other drug interactions are discovered only after analyzing the causes of treatment failure. To confirm the hypothesis that LOS and FUR can interact not only with HSA but also with each other, spectrophotometric measurements were performed. The mutual interactions were also analyzed by Momeni et al. and Ren et al. [30,31]. They studied the interaction between spermidine and bovine trypsin, as well as trypsin and resveratrol. Figure 5 presents the interaction between LOS and FUR, and the data were collected in Table 2.

Due to the interaction between the chemicals, the change in their absorption value is possible. If the absorbance of the mixture of two substances is different than the mathematic sum of two separate substances’ absorbance. Ren et al. wrote that this probably suggests the possibility of mutual interaction [31]. Based on the statistically significant changes in the absorbance values of LOS and FUR in the mixture (LOS + FUR) versus the mathematic sum of the mixture absorbance (Figure 5, Table 2), it can be concluded that LOS and FUR interact with each other. The most likely effect of the reaction between LOS and FUR is the formation of an ester bond due to the presence of hydroxyl group of LOS and the carboxyl group of FUR. 

### 2.2. Spectroscopic Analysis of LOS and FUR Influence on Albumin Secondary Structure

In order to estimate protein secondary structure in the presence of drugs (LOS, FUR), circular dichroism (CD) as an excellent tool for rapid determination of protein structure was used. As many authors described [32,33,34], the destructive effect of drugs on the secondary structure of protein is a common phenomenon. Table 3 and Table 4 present the value of HSA mean residue ellipticity [Θ]_MRW_ and the percentage (%) content of the secondary structure elements of HSA, respectively, both in the sample without drugs (HSA) and in the presence of drugs, in the binary LOS-HSA, FUR-HSA and ternary FUR-HSA_LOS_ and LOS-HSA_FUR_ systems.

On the basis of data collected in Table 3 and Table 4, it can be concluded that HSA is a α-helical protein. α-helix dominates in the serum albumin secondary structure, and the CD spectrum is characterized by a double minimum at 221 nm and 209 nm [35,36]. Due to the lack of significant changes between the values presented in Table 3 and Table 4, it can be concluded that the interactions between HSA and LOS/FUR, both separately and together, do not significantly affect the HSA secondary structure, regardless of the order of drugs administration used in the combination treatment. Moeinpour et al. have also observed that based on the DSSP (dictionary of secondary structure of proteins) method, LOS subtly affects the hydrogen bonds and thus the secondary structure of HSA [20]. The results obtained by Moeinpour et al. with the use of DSSP are significant, but due to the limitations of the applied method, they must be verified by experimentally obtained data, such as with the use of CD. Similar studies concerning the nonsignificant FUR influence on HSA secondary structure were obtained by Zaidi et al. [23] using HSA:FUR molar ratios 1:0 (control), 1:1 and 1:2, at 298 K, 303 K and 310 K. They analyzed a slightly higher HSA concentration than in the presented work and observed that with the increase of temperature (from 298 K to 310 K), the percentage of α-helical content decreases. A possibility of FUR impact on the secondary structure of proteins (human carbonic anhydrase II: hCAII) was also analyzed by Ranjbar et al. Using FUR concentrations 10, 20, 50 and 100 × 10^−6^ M (T = 298 K), they observed that binding of FUR to hCAII can change the percentage of α-helicity content of the protein from 9.87 ± 0.12% to 15.70 ± 0.10% [37].

### 2.3. Spectroscopic Analysis of LOS and FUR Influence on Albumin Antioxidant Activity

The use of both ABTS and DPPH assays methods to test the antioxidant activity of the analyzed samples is very important. It allows to obtain the information about HSA and ligands reaction with model free radicals as well as the effect of ligands-albumin binding in the presence of model free radicals on HSA tertiary structure. Moreover, using ethanol as DPPH solvent, it is possible to test the antioxidant activity of HSA under denaturing conditions, while ABTS assay allows for the analysis of HSA antioxidant activity under the native conditions (the reaction environment is phosphate buffer). 

Significant differences were observed between the results of DPPH and ABTS assays (Table 5, Table 6 and Table 7). There are many potential reasons for the observed differences, and the model free radicals used in both tests as well as the presence of ethanol in DPPH assay could play an important role. 

**Figure 6:**
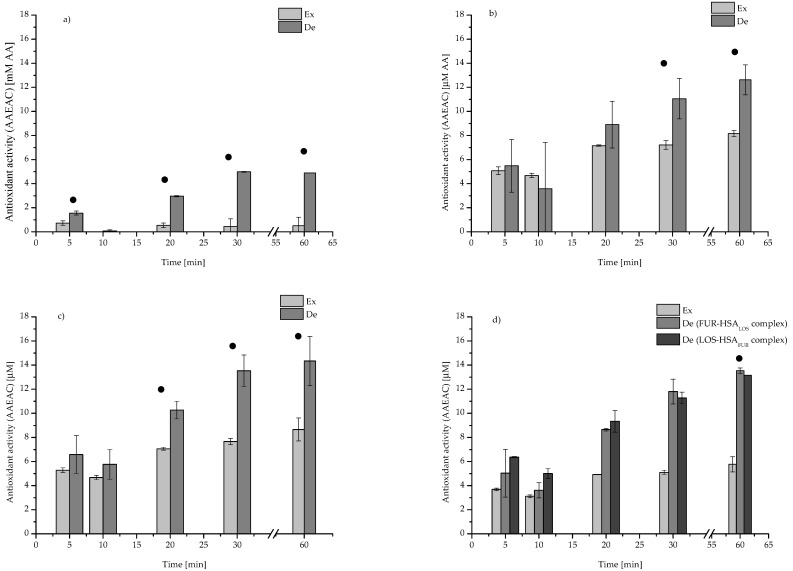
Expected versus designated values of antioxidant activity (AAEAC) of samples (**a**) LOS-FUR, (**b**) LOS-HSA (**c**) FUR-HSA, (**d**) FUR-HSA_LOS_ and LOS-HSA_FUR_ (DPPH assay); ●—statistically significant difference.

Using DPPH (Table 5 and Figure 6) and ABTS (Table 6 and Table 7), assays analyses of the antioxidant activity of HSA and of both LOS and FUR, separately and in the mixture, were conducted.

According to the data collected in Table 5 and on Figure 6, the antioxidant potential of all tested samples in denaturing conditions was the highest after 30 and 60 min of radical reaction initiation. For the samples of LOS and FUR solutions statistically significant changes in DPPH absorbance have not been observed. It probably means that between DPPH and LOS or FUR no reactions were observed. For the mixture of LOS and FUR (LOS-FUR at LOS:FUR 1:1 molar ratio), it was observed that the antioxidant potential was much higher than expected (Figure 6a), and it allows to conclude that as a result of mutual interaction between LOS and FUR, it is possible to reduce the level of DPPH.

Based on the data collected in Table 6 (ABTS assay), it can be stated that at 4 × 10^−4^ M concentration, both LOS and FUR scavenge the cationic ABTS radicals. FUR shows higher antioxidant activity than LOS. The value of AAEAC of sample with LOS-FUR (at LOS:FUR 1:1 molar ratio) mixture was close to zero, whereas the values of AAEAC of samples LOS and FUR in separate samples were 0.00 ± 0.00 [µM AA] and 2.61 ± 0.42 [µM AA], respectively. This in turn means that the antioxidant activity of the mixture of both drugs after 5 min, expressed with AAEAC, is significantly lower than the mean of AAEAC values for both drugs when they were in separate samples. After 5 min from the beginning of radical reaction initiation, an antagonistic effect between the LOS-FUR (LOS:FUR 1:1 molar ratio) mixture compared to LOS and FUR in separate samples was observed. After 30 and 60 min (for 4 × 10^−4^ M concentration of sample), a synergistic effect between the LOS-FUR mixture and ABTS was observed because the values of AAEAC of samples with LOS-FUR mixtures were significantly higher than the expected values of AAEAC. The expected values of AAEAC were calculated as the mean of AAEAC values for both drugs (LOS and FUR) after 30 and 60 min (Table 6). This probably means that the longer incubation time may result in more effective free radical scavenging by LOS and FUR in the mixture than in the separate samples. Similar conclusions were drawn by Bag et al. They have shown that the synergistic effect of coriander/cumin (Coriander Rf: 0.35 + Cumin Rf: 0.53) against DPPH can be observed after 30 min of incubation [9]. The activity of LOS can be compared with another drug. This concept was used, e.g., by Gheitasi et al. They analyzed the effect of therapy with LOS and α-tocopherol during the course of “acute ureteral obstruction-induced renal excretory dysfunction and acidification defect” in Sprague-Dawley rats. They showed that both LOS and LOS with α-tocopherol can have a statistically significant influence on the antioxidant activity of urine (on the basis of FRAP assay) [38]. Similarly, FUR used in low doses during therapy together with propranolol may significantly increase the activity of enzymatic antioxidant factors (glutathione reductase and glutathione peroxidase) as well as the level of reduced glutathione [39]. Importantly, the hydroxyl group of LOS and the carboxyl group of FUR are important in reactions with free radicals. This probably explains why the antioxidant activity of the LOS and FUR mixture is different than the mathematic sum of the LOS and FUR (separately) antioxidant activities. 

The influence of LOS on the antioxidant activity of specific biological samples (plasma, urine) was studied in vivo and in vitro by Ivanov et al. [40] and Lin et al. [41]. They observed that LOS may show the ability to increase catalase and glutathione peroxidase activities, and it also reduces the level of lipid peroxidation [40,41]. A possible consequence of LOS therapy with Wistar diabetic rats is also a reduction the oxidative injury of renal DNA. Furthermore, Lodovici et al. indicated that during the treatment with the use of LOS, the level of antioxidant activity in the plasma of diabetic rats was similar to the level recorded for healthy rats [42]. Kayabasi et al. observed that LOS significantly reduces oxidative stress and contributes to reducing the negative effects of free radical activity. The supply of LOS caused not only an increase of plasma antioxidant activity in patients suffering from end-stage renal disease (ESRD) but also led to an increase in the level of free thiol groups [43]. Karanovic et al. showed that hypertension and chronic kidney disease (CKD) rats’ treatment with LOS (using ABTS assay) can lead to an increase in plasma antioxidant activity [44]. This in turn means that it is reasonable to investigate whether the beneficial effect of LOS on plasma antioxidant activity as well as the influence of LOS on the level of free thiol groups is related to its interaction with HSA. However, using a DPPH assay, Teixeira et al. showed LOS low antioxidant activity [45]. Despite the fact that they applied the methanolic solutions of reagents, as well as other than in the present work DPPH:samples volume ratio (*v*:*v*), the results are comparable to those presented in this study, and the use of an alternative method of analysis such as ABTS is necessary. 

FUR antioxidant activities were well described by Lahet et al. [46], and analyzing the in vitro studies (Allophycocyanin assay), the increase of FUR concentration with the increase of analyzed samples antioxidant potential was identified while in vivo studies (Wistar rats) showed the influence of FUR on blood from abdominal aorta antioxidant activity [46]. It is noteworthy that through the studies of Lahet et al., furosemide was selected as the second (first was LOS) drug with the ability to scavenge model free radicals (DPPH and ABTS), and it also may be a modulator or co-modulator of HSA antioxidant activity. 

The binding of HSA by various ligands has an influence on the course of the reaction between DPPH and HSA. This phenomenon is especially noticeable, when ligands such as ascorbic acid, α-tocopherol, melatonin or β-carotene are strong antioxidants [8]. Cao et al. studied the incubation of various polyphenols with bovine serum albumin (BSA), and they observed that the 7-day incubation caused a higher antioxidant potential in polyphenols relative to DPPH than that in samples without BSA [7]. There is some relationship between the affinity of ligands for protein and the antioxidant potential of the ligand–protein complex. In the present work, we hypothesize that also weak antioxidants such as LOS and FUR can significantly affect the reaction between HSA and DPPH or ABTS. 

The designated antioxidant activity of protein (DPPH assay) in LOS-HSA (Figure 6b) complex is higher than expected after 30 and 60 min from the beginning of the radical reaction initiation, and the designated antioxidant activity of HSA in the FUR-HSA (Figure 6c) complex is higher than expected after 20, 30 and 60 min from the beginning of the radical reaction initiation. A similar tendency was observed after 20 min for FUR-HSA_LOS_ and LOS-HSA_FUR_ complexes, and the order of drugs administration was irrelevant (Figure 6d). These results were much lower than in the presence of ABTS. Cationic ABTS radicals were removed very fast by HSA, both in the absence and in the presence of drugs. The analysis of antioxidant activity was possible only after 5 and 10 min from the beginning of the radical reaction initiation. Similar results, concerning HSA high antioxidant activity against the cationic radical ABTS, were obtained by Ihara et al. [47]. Between 20 and 60 min from the beginning of reaction, a very high antioxidant activity of analyzed substances was registered (Table 7). As it was previously written, one of the main roles of HSA is regulation of the level of free radicals and cysteinyl residue Cys-34, and Met-87, Met-123, Met-298, Met-329, Met-446 and Met-548 are mainly responsible for protein antioxidant activity [1,48,49,50,51]. Due to the fact that Cys-34 is located in domain I (subdomain IA) on the surface of HSA and has only one free thiol group, its modification as well as change of location (as a result of conformational changes) may affect the ability to free radical scavenging by HSA [1,52,53]. Close to Cys-34, three other amino acid residues (Asp-38, His-39 and Tyr-84) are also located and may regulate its activity [49].

Regardless the ligand (LOS or FUR) and the order of their administration, this process is accompanied by a synergistic effect, and this type of interaction effect between HSA and analyzed ligands was observed, when only HSA antioxidant activity was shown (the concentration of the samples: LOS and FUR 1 × 10^−4^ M, HSA 0.5 × 10^−4^ M). Based on this investigation, it can be concluded that the binding of LOS and FUR by HSA does not disturb the antioxidant activity of this protein. Taking into account the obtained results, it can be concluded that the increase in the free radical scavenging capacity of HSA by binding with LOS and FUR, both in the binary and ternary systems, probably means that ligands contribute to increase Cys-34 exposure.

## 3. Materials and Methods

### 3.1. Chemicals

Losartan (LOS) was purchased from Biofarm sp. z o. o. Human serum albumin (HSA), factor V Lot No. 4971K and furosemide (FUR) Lot No. 2508J were purchased from MP Biomedicals. 2,2′-Azino-bis(3-ethylbenzothiazoline-6-sulfonic acid) diammonium salt (ABTS) Lot No. SLBZ8095 and 2,2-Diphenyl-1-picrylhydrazyl (DPPH) Lot No. STBH727 were from Sigma Aldrich, potassium persulfate (K_2_S_2_O_8_) and ascorbic acid (C_6_H_8_O_6_) from Chempur while di-Potassium hydrogen phosphate pure p.a. (K_2_HPO_4_) and sodium dihydrogen phosphate dihydrate (NaH_2_PO₄ × 2H_2_O) from Eurochem BGD Sp. z o. o. All chemicals were of analytical grade and used without further purification. 

### 3.2. Methods

#### 3.2.1. Nano Isothermal Titration Calorimetry (nanoITC)

Calorimetric measurements were carried out using nanoITC instrument (TA Instruments, New Castle, DE, USA). All samples were prepared, stored and tested at room temperature. Using Degassing Station (TA Instruments, New Castle, DE, USA) the samples were degassed (t = 20 min). Initial cell volume was 300 µL; injection intervals were 180 s; injection volume was 2.38 µL, and stir rate was 300 rpm. Human serum albumin, losartan, furosemide and phosphate buffer (pH 7.4) concentrations were 3 × 10^−5^ M, 3.25 × 10^−3^ M, 8.1 × 10^−4^ M and 5.0 × 10^−2^ M, respectively. As blank (by injection) phosphate buffer was used. As a reference system for the test samples a HSA solution has been used. Phosphate buffer (0.05 M; pH 7.4) was prepared by mixing of 1.56 g NaH_2_PO₄ × 2H_2_O and 6.96 g K_2_HPO_4_ in 1 dm^3^ of distilled water. All measurements with the use of nanoITC were carried out at the temperature of 298 [K]. All results were performed with the use of NanoAnalyze Data Analysis Version 3.10.0 (TA Instruments, New Castle, DE, USA). 

The Gibbs free energy change ΔG was obtained based on the Equation (1) [25]:

∆G = ∆H − T∆S(1)

where:

ΔG—Gibbs free energy change [kcal/mol];
T—temperature [K];
ΔS—entropy change [kcal/molK];
ΔH—enthalpy change [kcal/mol].

#### 3.2.2. UV–VIS Spectrophotometry Measurements 

Absorption measurements and antioxidant activity studies were carried out using JASCO V-730 UV-Visible spectrophotometer (JASCO International CO., LTD., Hachioji, Tokyo, Japan). The absorption spectra of LOS and FUR at concentration 1 × 10^−5^ M and LOS-FUR in the system at LOS:FUR 1:1 (*v*/*v*) molar ratio, were determined in the wavelength range from 200 to 350 nm with 10 mm path length quartz cuvettes. 

Based on the protocol described previously, DPPH and ABTS assays were used to test the antioxidant activity of the samples [54]. DPPH (2,2-difenylo-1-pikrylohydrazyl) is a free radical with purple color in ethanolic solution, and under the influence of antioxidants, the DPPH solution becomes discolored. The concentration of HSA was 2 × 10^−4^ M and LOS:HSA and FUR:HSA molar ratios were 2:1. Protein solution as well as ligands solutions were mixed in volume ratio (*v*/*v*) 1:1. DPPH solution at 1 × 10^−4^ M concentration was mixed with the samples in volume ratio (*v*/*v*) 1:1, and the maximum absorption of DPPH at 517 nm was registered after 5, 10, 20, 30 and 60 min. 

ABTS (2,2′-azino-bis(3-ethylbenzothiazoline-6-sulfonic acid) can create a cationic radical as a result of the reaction with potassium persulfate. It has an intense green color, and due to the reaction with antioxidant substances, its discoloration occurs. The concentrations of ligands (LOS as well as FUR) were 4 × 10^−4^ M and 1 × 10^−4^ M; the concentration of HSA was 5 × 10^−5^ M, and the concentration of LOS and FUR in the system with HSA was 1 × 10^−4^ M. The LOS:HSA and FUR:HSA molar ratio was 2:1. ABTS and potassium persulfate solutions at concentrations 5 × 10^−3^ M and 1.74 × 10^−3^ M, respectively, were incubated at room temperature in the dark (16 h). After the incubation time, ABTS reagent was mixed with the samples at volume ratio (*v*/*v*) 1:1, and the cationic radical ABTS maximal absorbance at 734 nm was measured after 5, 10, 20, 30 and 60 min. 

The % inhibition value was calculated on the basis of the following Equation (2) [55,56]:

(2) $$\%\;\mathrm{inhibition}=\left(\frac{A_{0}-A_{1}}{A_{0}}\right)\;\times \;100\%\;$$

where:

A_0_, A_1_—the initial absorbance of DPPH or ABTS, in the absence and presence of the samples, respectively.

In order to compare the obtained results with the source data, the % inhibition values (DPPH and ABTS assay) were converted into the concentration of ascorbic acid (from 2.22 × 10^−6^ M to 3.548 × 10^−5^ M), and a value of Total Antioxidant Capacity (AAEAC, Ascorbic Acid Equivalent Antioxidant Capacity) was determined.

#### 3.2.3. Circular Dichroism (CD) Measurements

CD spectrum of HSA was measured using Jasco J-1500 spectropolarimeter (JASCO International CO., LTD., Hachioji, Tokyo, Japan). The spectra were registered in the wavelength range between 200 and 250 nm, the wavelength intervals 0.5 nm, 1 mm path length quartz cuvette. Preparation, storage and testing of samples were performed at room temperature with the use of thermostatic Peltier cell holder, with an accuracy of ±0.05 °C. Human serum albumin, ligands (LOS, FUR) and phosphate buffer (pH 7.4) concentrations were 3.0 × 10^−6^ M, 4.0 × 10^−4^ M and 0.05 M, respectively. 

The mean residue ellipticity [Θ]_MRW_ was calculated using the Equation (3) [35,57]:

(3) $$\left[\Theta {]}_{\mathrm{MRW}}=\frac{\mathrm{MRW}\;\times \;\Theta }{\begin{array}{c} 10\times l\times m \end{array}}\;[\deg\;\times \;{\mathrm{cm}}^{2}\;\;{\mathrm{dmol}}^{-1}\right]$$

where:

Θ—observed ellipticity for a given wavelength [deg]
m—the concentration [g/cm^3^]
l—the pathlength [cm]
MRW—a mean residue weight (MRW _HSA_ = 113.7 Da).

### 3.3. Statistics 

All results were expressed as a mean ± relative standard deviation (SD) from minimum two independent experiments. In order to analyze the obtained results OriginPro Software Version 8.5 SR1 (OriginLab Corporation, Northampton, MA, USA), Microsoft Excel 2013 (Microsoft Corporation, Redmond, WA, USA), Statistica (data analysis software system), version 13; (TIBCO Software Inc. 2017, Palo Alto, CA, USA) as well as Spectra Manager Version 2.13.00 2002–2015 (JASCO International Co., Ltd., Hachioji, Tokyo, Japan) were used. 

## 4. Conclusions

The losartan (LOS) as well as furosemide (FUR), angiotensin II receptor blockers, separately as well as in the presence of model free radicals, DPPH and ABTS, were studied in terms of the analysis of antioxidant activity. The mutual interaction at the molecular level between LOS and FUR was identified. Both drugs showed a high affinity for HSA. It was identified based on the Gibbs free energy ΔG and enthalpy ΔH changes. The binding reaction between protein and ligands was spontaneous with exoenergetic nature (ΔG < 0, ΔH < 0). The interaction between LOS and HSA was accompanied by the ionic bonds (electrostatic forces) (ΔS > 0, ΔH ≤ 0) while in the FUR-HSA complex, van der Waals forces and hydrophobic interaction dominated (ΔS < 0, ΔH < 0). LOS, FUR and their mixture do not significantly impact on the secondary structure of HSA. Moreover, LOS and FUR, separately and simultaneously, modulate the antioxidant activity of HSA, and the interactions are synergistic. 

The simultaneous use of losartan and furosemide in patients does not adversely affect the antioxidant activity of HSA. Both of these drugs can help to increase the effectiveness of the elimination of free radicals from the human body.

## Figures and Tables

**Table 1:** Drugs–albumin interaction parameters. T = 298 [K].

Parameters	LOS	FUR
K_a_ ± SD * × 10^4^ [M^−1^]	6.22 ± 1.96	4.11 ± 1.22
n ± SD *	6.16 ± 0.33	1.73 ± 1.18
ΔH ± SD * [kcal/mol]	−1.10 ± 0.01	−11.54 ± 3.77
ΔS ± SD * [cal/molK]	18.21 ± 0.67	−17.65 ± 12.06
ΔG ± SD * [kcal/mol]	−6.52 ± 0.19	−6.28 ± 0.18

* SD—standard deviation.

**Table 2:** The average values of maximum absorbance of LOS, FUR and drugs mixture at the maximum wavelengths λ_max (LOS)_ 207 nm, λ_max (FUR I)_ 230 nm, λ_max (LOS + FUR I)_ 218 nm, λ_max (FUR II)_ 278 nm.

λ_max_ [nm]	Absorbance ± SD *			Mathematic Sum of LOS and FUR Absorbance ± SD *	Effect ** (I/NI)
	LOS	FUR	LOS + FUR		
207	0.4938 ± 0.0009	0.3155 ± 0.0017	0.7436 ± 0.0027	0.8092 ± 0.0027	I
218	0.3514 ± 0.0004	0.3523 ± 0.0041	0.6709 ± 0.0045	0.7038 ± 0.0045	I
230	0.2626 ± 0.0002	0.4165 ± 0.0010	0.6576 ± 0.0012	0.6791 ± 0.0012	I
278	0.0475 ± 0.0005	0.3021 ± 0.0103	0.3187 ± 0.0110	± 0.0108	I

* SD—standard deviation. ** I—interaction; NI—no interaction.

**Table 3:** CD spectra and the values of HSA mean residue ellipticity [Θ]_MRW_ in the sample without drugs (HSA) and in the presence of drugs, in the binary LOS-HSA and FUR-HSA and ternary FUR-HSA_LOS_ and LOS-HSA_FUR_ systems.

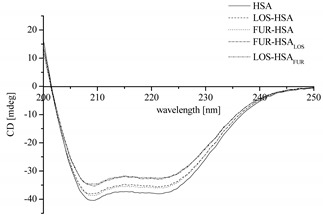		
Sample	λ_min_ [nm]	[Θ]_MRW_ ± SD * [deg × cm^2^ × dmol^−1^]
HSA (3.0 × 10^−6^ M)	209	−22,867.66 ± 218.75
	221	−21,543.93 ± 201.70
LOS-HSA (molar ratio 4.44:1)	209	−21,882.95 ± 316.43
	221	−20,584.20 ± 363.67
FUR-HSA (molar ratio 4.44:1)	209	−21,969.26 ± 51.14
	221	−20,580.90 ± 92.13
FUR-HSA_LOS_ (molar ratio 4.44:1:4.44)	209	−19,931.44 ± 131.10
	221	−18,647.65 ± 180.95
LOS-HSA_FUR_ (molar ratio 4.44:1:4.44)	209	−19,713.56 ± 41.71
	221	−18,498.56 ± 51.83

* SD—standard deviation.

**Table 4:** The percentage (%) content of HSA secondary structure elements in the sample without drugs (HSA) and in the presence of drugs, in the binary LOS-HSA and FUR-HSA and ternary FUR-HSA_LOS_ and LOS-HSA_FUR_ systems (Yang’s reference model).

Sample	% α-Helix ± SD *	% β-Sheet ± SD *	% Turn ± SD *	% Other ± SD *
HSA (3.0 × 10^−6^ M)	36.55 ± 1.06	11.95 ± 0.64	20.75 ± 0.07	30.70 ± 0.42
LOS-HSA (molar ratio 4.44:1)	35.70 ± 0.14	12.60 ± 0.14	20.70 ± 0.00	31.05 ± 0.07
FUR-HSA (molar ratio 4.44:1)	35.90 ± 0.42	13.20 ± 0.00	20.40 ± 0.28	30.45 ± 0.21
FUR-HSA_LOS_ (molar ratio 4.44:1:4.44)	35.70 ± 0.42	12.95 ± 0.49	20.75 ± 0.21	30.60 ± 0.14
LOS-HSA_FUR_ (molar ratio 4.44:1:4.44)	35.20 ± 0.00	13.35 ± 0.21	20.40 ± 0.00	31.10 ± 0.14

* SD—standard deviation.

**Table 5:** The Total Antioxidant Capacity (AAEAC) (DPPH assay).

Antioxidant Activity (AAEAC) ± SD * [µM AA]					
Sample	Time [min]				
	5	10	20	30	60
HSA (2 × 10^−4^ M)	9.63 ± 0.05	9.35 ± 0.36	13.68 ± 0.36	14.43 ± 0.72	16.32 ± 0.48
LOS (4 × 10^−4^ M)	0.50 ± 0.71	0.00 ± 0.00	0.63 ± 0.25	0.00 ± 0.00	0.00 ± 0.00
FUR (4 × 10^−^^4^ M)	0.94 ± 0.34	0.00 ± 0.00	0.44 ± 0.14	0.88 ± 1.25	1.00 ± 1.41
LOS-FUR (molar ratio 1:1)	1.55 ± 0.17	0.07 ± 0.09	2.96 ± 0.05	4.98 ± 0.03	4.89 ± 0.01
LOS:HSA (molar ratio 2:1)	5.48 ± 2.19	3.58 ± 3.86	8.91 ± 1.94	11.05 ± 1.68	12.62 ± 1.25
FUR:HSA (molar ratio 2:1)	6.59 ± 1.57	5.77 ± 1.24	10.27 ± 0.73	13.52 ± 1.32	14.34 ± 2.04
FUR-HSA_LOS_ (molar ratio 2:1:2)	5.04 ± 1.98	3.62 ± 0.63	8.65 ± 0.11	11.80 ± 1.03	13.53 ± 0.23
LOS-HSA_FUR_ (molar ratio 2:1:2)	6.37 ± 0.05	5.01 ± 0.42	9.33 ± 0.89	11.27 ± 0.48	13.15 ± 0.00

* SD—standard deviation.

**Table 6:** The Total Antioxidant Capacity (AAEAC) (ABTS assay).

	Antioxidant Activity (AAEAC) ± SD * [µM AA]				
Sample	Time [min]				
	ABTS Assay				
	5	10	20	30	60
LOS (1 × 10^−4^ M)	0.00 ± 0.00	0.00 ± 0.00	0.00 ± 0.00	0.00 ± 0.00	0.00 ± 0.00
FUR (1 × 10^−4^ M)	0.00 ± 0.00	0.00 ± 0.00	0.00 ± 0.00	0.00 ± 0.00	0.44 ± 2.86
LOS:FUR (molar ratio 1:1)	0.00 ± 0.00	0.00 ± 0.00	0.00 ± 0.00	0.00 ± 0.00	0.00 ± 0.00
LOS (4 × 10^−^^4^ M)	0.00 ± 0.00	0.46 ± 0.37	1.53 ± 0.54	2.19 ± 0.59	2.85 ± 0.61
FUR (4 × 10^−^^4^ M)	2.61 ± 0.42	5.48 ± 0.55	7.73 ± 0.01	9.67 ± 1.13	12.18 ± 2.81
LOS:FUR (molar ratio 1:1)	0.16 ± 0.23	2.08 ± 0.16	4.51 ± 0.26	6.95 ± 0.42	8.83 ± 1.41

* SD—standard deviation.

**Table 7:** Expected versus designated values of antioxidant activity AAEAC of samples at LOS:HSA = FUR:HSA = 2:1 and LOS:FUR:HSA = FUR:LOS:HSA = 2:2:1 molar ratios (ABTS assay).

	Antioxidant Activity (AAEAC) ± SD * [µM AA]					
Sample		Time [min]				
		ABTS Assay				
		5	10	20	30	60
HSA (5 × 10^−5^ M)	De	23.85 ± 0.24	25.57 ± 0.43	27.36 ± 0.35	27.09 ± 0.63	26.01 ± 0.12
LOS-HSA (molar ratio 2:1)	De	17.07 ± 0.51	22.31 ± 0.56	25.50 ± 0.50	26.00 ± 0.37	25.72 ± 0.30
	Ex	11.93 ± 0.12	12.78 ± 0.22	13.68 ± 0.17	13.55 ± 0.31	13.01 ± 0.06
	Effect	s	s	s **	s **	s **
FUR-HSA (molar ratio 2:1)	De	19.19 ± 0.02	24.02 ± 0.16	26.60 ± 0.12	25.75 ± 1.61	26.00 ± 0.20
	Ex	11.93 ± 0.12	12.78 ± 0.22	13.68 ± 0.17	13.55 ± 0.31	13.12 ± 0.09
	Effect	s	s	s **	s **	s **
FUR-HSA_LOS_ (molar ratio 2:1:2)	De	12.41 ± 0.45	18.32 ± 0.11	21.72 ± 0.67	21.58 ± 1.81	24.37 ± 1.96
	Ex	7.95 ± 0.08	8.52 ± 0.14	9.12 ± 0.12	9.03 ± 0.21	8.74 ± 0.14
	Effect	s	s	s **	s **	s **
LOS-HSA_FUR_ (molar ratio 2:1:2)	De	12.43 ± 0.64	17.82 ± 0.33	22.11 ± 0.42	24.37 ± 1.96	24.59 ± 0.45
	Ex	7.95 ± 0.08	8.52 ± 0.14	9.12 ± 0.12	9.03 ± 0.21	8.74 ± 0.14
	Effect	s	s	s **	s **	s **

* SD—standard deviation. N/A—not applicable. Ex—expected. De—designated. ad—an additive effect: expected and designated values reveal lower differences than 5%; s—a synergistic effect: designated values are more than 5% higher for AAEAC when compared with expected values. an—an antagonistic effect: designated values are more than 5% lower for AAEAC when compared with expected values [6]. ** the most likely effect, as a result of the very strong antioxidant activity of HSA in relation to ABTS, it is very difficult to clearly define the observed interaction effect.

## Data Availability

Data is contained within the article.
